# Putting Proteomics Into Immunotherapy for Glioblastoma

**DOI:** 10.3389/fimmu.2021.593255

**Published:** 2021-02-23

**Authors:** Liangyu Chen, Di Qin, Xinyu Guo, Qixue Wang, Jie Li

**Affiliations:** ^1^Department of Proteomics, Tianjin Enterprise Key Laboratory of Clinical Multi-omics, Tianjin, China; ^2^Department of Neurosurgery, Tianjin Medical University General Hospital, Tianjin, China; ^3^Laboratory of Neuro-Oncology, Tianjin Neurological Institute, Tianjin, China

**Keywords:** glioblastoma, proteomics, heterogeneous microenvironment, immunotherapy, biotarget

## Abstract

In glioblastoma, the most aggressive brain cancer, a complex microenvironment of heterogeneity and immunosuppression, are considerable hurdles to classify the subtypes and promote treatment progression. Treatments for glioblastoma are similar to standard therapies for many other cancers and do not effectively prolong the survival of patients, due to the unique location and heterogeneous characteristics of glioblastoma. Immunotherapy has shown a promising effect for many other tumors, but its application for glioma still has some challenges. The recent breakthrough of high-throughput liquid chromatography–mass spectrometry (LC-MS/MS) systems has allowed researchers to update their strategy for identifying and quantifying thousands of proteins in a much shorter time with lesser effort. The protein maps can contribute to generating a complete map of regulatory systems to elucidate tumor mechanisms. In particular, newly developed unicellular proteomics could be used to determine the microenvironment and heterogeneity. In addition, a large scale of differentiated proteins provides more ways to precisely classify tumor subtypes and construct a larger library for biomarkers and biotargets, especially for immunotherapy. A series of advanced proteomic studies have been devoted to the different aspects of immunotherapy for glioma, including monoclonal antibodies, oncolytic viruses, dendritic cell (DC) vaccines, and chimeric antigen receptor (CAR) T cells. Thus, the application of proteomics in immunotherapy may accelerate research on the treatment of glioblastoma. In this review, we evaluate the frontline applications of proteomics strategies for immunotherapy in glioblastoma research.

## Introduction

Glioblastoma is one of the top malignant brain cancers. Standard therapies only result in poor prognosis and low survival rates. Novel treatment approaches are desperately needed. Subtype classification is very important for precision medicine of cancer treatment to achieve a better prognosis. Even though advanced nucleic acid technology together with other clinical features have made considerable progress in this step for glioblastoma, the heterogeneous characteristics still cannot be overcome.

The standard care for glioblastoma is similar to that of other cancers, but due to the special location of glioblastoma and its heterogeneity, standard therapies do not turn out the ideal prognosis for glioblastoma. The appearance of immunotherapy provided a more specific and efficient approach to prolong the survival of patients with cancer. Several different strategies have been proposed to target different parts of the tumor. However, heterogeneity again makes it difficult to apply single or several existing immunotherapy methods to yield better consequences in glioblastoma. There are several challenges facing immunotherapy for glioblastoma. A more complicated mechanism needs to be elucidated to identify more useful biomarkers and biotargets, which has been almost beyond the ability of many prime research methods. Efficient evaluation methods are also necessary for immunotherapy.

Proteomics, which has been developing rapidly in the last decade, is important for whole-tumor research. Compared with whole-genome sequencing or transcriptome sequencing, which can only indicate the origin of tumors, proteomics can reveal the actual state of tumor cells by quantifying functional proteins, the cell function operators. High-throughput mass spectrometry (MS) technology can be used to evaluate tumors with higher dimensions. The ability to quantify thousands of proteins at the same time simplifies the process of studying the mechanisms of tumor development and can filter certain biomarkers and target candidates. Thus, the application of proteomics can enhance the efficiency of glioblastoma research. In particular, single-cell proteomics has also provided an even more specific tool to investigate the heterogeneous microenvironment of glioblastoma. In this review, we discuss immunotherapy for glioblastoma and its challenges, and proteomics methods are presented and shown as applications for solving these challenges.

## Glioblastoma

Glioma is responsible for 27% of all central nervous system (CNS) tumors and 80% of malignant tumors (1) occurring among people aged from 15 to 34 years around the world. About 2.5% of cancer-related death is caused by malignant gliomas (2). In 2016, the WHO classified gliomas into three main types based on histological methods: astrocytoma, oligodendroglioma, and oligoastrocytoma (3, 4). Later, the newly published World Health Organization Classification of Tumors of the Central Nervous System (WHO CNS 2016) further classified tumors as WHO I–IV based on the combination of both histological and molecular information (5). Patients categorized under WHO IV had the most malignant degree of tumors, which were called high-grade colloid tumors or glioblastoma (6).

Glioblastoma is mostly diagnosed as primary glioblastoma (*de novo*) and is more common in elderly patients. Astrocytomas would transform to a malignant tumor to become the source of some secondary glioblastomas (7). From a microscopic view, glioblastoma is characterized by growth and morphology, including cell number, anaplasia, mitotic activity, and microvascular condition (8). Besides the histological information, the mutations of genes, IDH, ATRX, TERT, EGFR, MGMT, etc., have all been included to further diagnose the subtypes of glioma or predict progress benefit. Specifically, for glioblastoma, EGFR, TERT, and +7/−10 cytogenetic signature are the molecular markers, and MGMT is a predictive biomarker of the benefit from alkylating chemotherapy (9).

## Advances in Proteomics for Glioma

“Proteome” is a word combining “protein” and “genome” that was proposed by Wilkins in 1994. Proteome refers to all the proteins in cells, tissues, or even in creatures and is extraordinarily complicated. Proteomics is a new technology to identify and analyze all the proteins present in biological samples from a holistic perspective. Proteomics can study the expression of proteins and the interaction between proteins. With the fast development of equipment and software, the most advanced proteomics techniques are based on MS and can be generally put into two categories: bottom-up proteomics (BUP) and top-down proteomics (TDP) (10). BUP differs from TDP in the prior steps of enzyme digestion of proteins to peptides and the liquid chromatography–mass spectrometry (LC-MS) separation and analysis. High-throughput MS systems make it possible to identify thousands of proteins at one time. Consequently, proteomics has become a more important technology to study the omics of different creatures and a powerful tool to research the mechanisms of tumor development to locate treatment markers. The proteomic strategy can be easily applied to the research of natural production mechanism in plants (11) or microorganism (12). This strategy has also been successfully applied to different types of diseases, such as Alzheimer's (13), periodontal disease (14), or thyroid-related diseases (15) and various kinds of tumors.

Since 2016, glioblastomas have mainly been classified based on the molecular genetic properties accompanied by other features (16). The ideal marker should not only be 100% sensitive, specific, and efficient for detection but should also be easily accessible for analysis and provide a simple analytical method and accurate information (17). Various biomarkers have been applied for different types of tumors. For glioblastoma, microRNAs (miRNAs), small molecules, circulating tumor cells (CTCs), extracellular vesicles, tumor tissues, and biological fluids are the most widely used besides nucleic acid and proteins. Unlike gene markers, which only indicate the possibility of having a type of tumor, identified proteins would confirm what is ongoing in the tissue and further divide tumors into more specific subtypes.

Proteins are becoming diagnostic and prognostic markers in different tumors including glioblastoma. Proteins are widely located in cancer tissues (18) and liquid matrices such as blood (19) and cerebrospinal fluid (CSF) (20). Though studies have verified that certain nucleic acids are more specific than other features including proteins, the breakthrough of MS technology has made proteins a strong assistant method. As nucleic acids cannot be used to evaluate the specific situation of tumors in cancer development, the combination of gene expression and proteomics is still necessary (20). Full proteomics tumor profiles would compare both natural and posttranslational changes during cancer development so that the mechanism of tumor development would be more specifically elucidated (2). For instance, the proteomic has been integrated with other methods to research on Pediatric Brain Cancer to explore novel biomarkers in recently published research (21). The study of protein posttranslational modifications could lead to the discovery of novel biomarkers and novel strategies for treatment (22).

In view of the possible lack of specificity of protein markers, a multiparameter comprehensive evaluation method was proposed, which is a combination of qualitative and quantitative analysis of several different protein markers to simultaneously filter the misleading false results in the identification of proteins (20, 23). This multiparametric evaluation can not only distinguish healthy or ill patients but also allow the diagnosis of specific tumor subtypes (20, 24, 25). A low concentration of proteins in biological fluid samples might be the most important problem. Moreover, extensive validation is still required when using proteins as biomarkers due to their heterogeneous nature (26). For instance, different glioblastoma cells with different microenvironments exhibit different *in vitro* invasion and cell migration abilities (27).

### Proteomics Strategy

#### Bottom-Up Strategy for Proteomics

**Figure 1:**
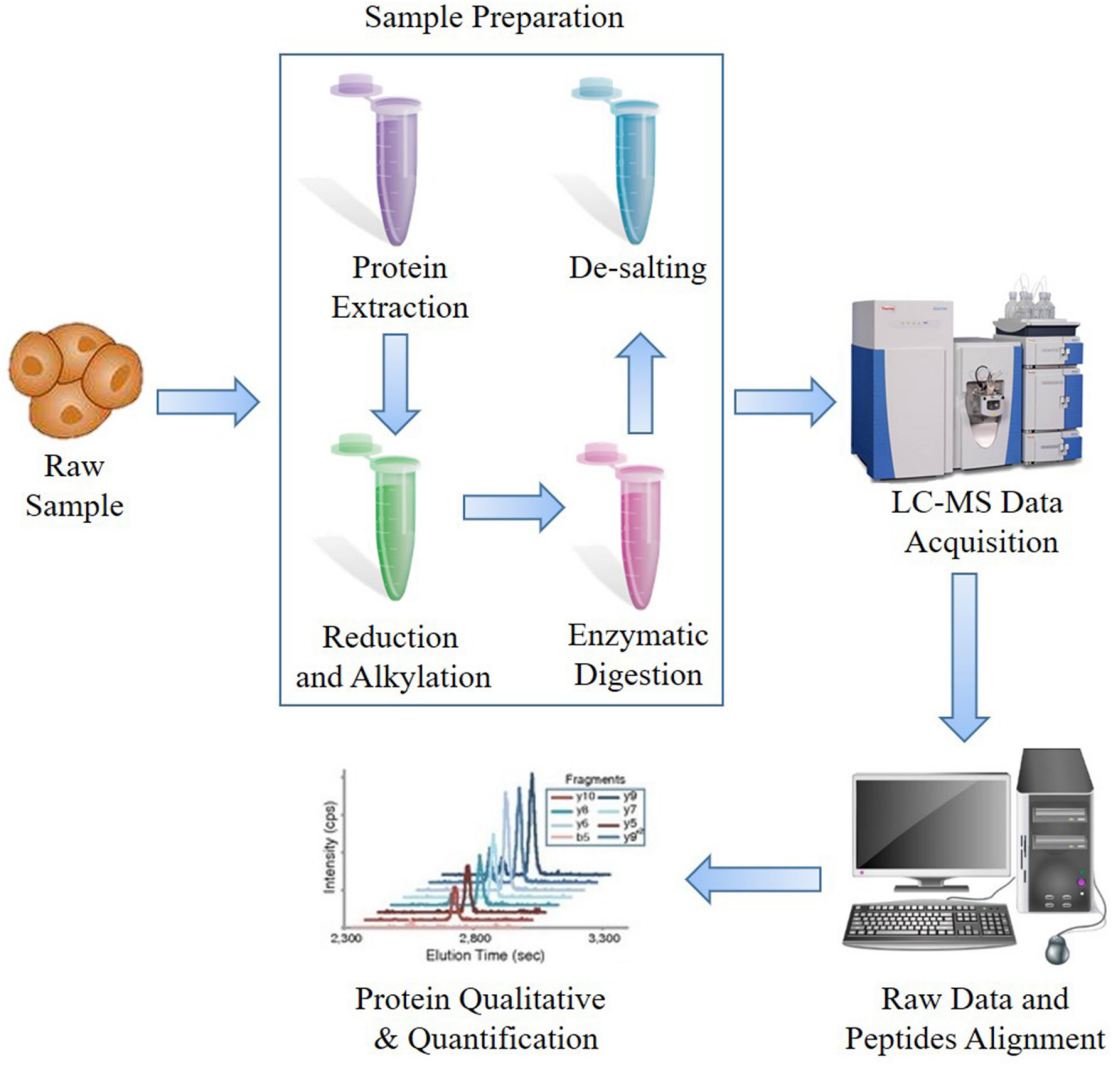
General procedure of bottom-up proteomics (BUP) mass spectrometry (MS)-based proteomics.

The prime procedure and most widely used strategy are BUP, which is performed from peptides (bottom) to proteins (up) (Figure 1). Generally, the proteins would be extracted from samples and then digested into peptides, and then the peptides would be purified and detected by LC-MS system to acquire peptide ion information, which is assembled and analyzed using specific software. The majority of researches on microorganisms, plants, or animals have regarded BUP as a prime option. Typically, BUP applies enzymes to cleave extracted mixed proteins from collected samples, including formalin-fixed and paraffin-embedded (FFPE), tissues or cultured cells, to small peptides of ~6–50 amino acids, which are optimum for MS detection and computational analysis (10). Trypsin is one of the most commonly used enzymes for an average output length of ~14 amino acids (28). The advantages of small peptide fragments are that they increase the separation efficiency, avoid the inability to detect isotopic peaks of proteins, and lighten the burden of searching through a database and assigning them to certain proteins. However, there is a key limitation of BUP that when the proteins are turned into fragments, the information regarding the proteoform, including the location and number of posttranslational modifications (PTMs) and endogenous proteolysis is lost (29). Furthermore, due to the increased complexity of mixtures of peptides, only some peptides can be detected, and the coverage of the assembled protein sequences is normally under 20%. To compensate for the shortcomings of BUP, the middle-down strategy was proposed such that the proteins could be digested into longer peptides and then sequenced.

Higher resolution and throughput would cover more peptides and identify more proteins. The newly applied orbitrap technology developed by Thermo Fisher (30) boosts the coverage and efficiency of BUP. Considering the large quantities of MS data, a powerful software and a complete database are necessary. The major companies involved in the proteomics business have developed their own systems to assist with their equipment. Other platforms such as Spectronaut, Peaks, MaxQuant (31), and Skyline (32) might be widely chosen by many researchers. However, as open databases are quite limited and the MS data from different types of machines might differ from each other, this is a great obstacle for research exchange between different labs.

#### Top-Down Strategy for Proteomics

On the other hand, a novel developed strategy, TDP, is becoming available. TDP aims to separate protein mixtures first and then sequence the intact proteins. Thus, the protein sequences from the TDP strategy would mostly be 100% complete, and even the PTMs of proteins with the same sequences could be distinguished. This could provide a deeper understanding of proteoforms *in vivo* (33). The three typical steps are as follows: separation of the protein mixture; detection of the molecular weight by MS; and data processing and database searching/scoring (34).

Multiple methods have been proposed to improve the sample condition before MS in the first step. Hydrophilic interaction liquid chromatography (HILIC) (35), weak cation exchange (WCX) (36), capillary reversed-phase liquid chromatography (RPLC) (37), and capillary isoelectric focusing (CIEF) (38) are typical representative on-line technologies that are used before MS detection. High sensitivity, high resolution, and high throughput are necessary for the sequencing of mixed proteins with a large m/z. Thus, Fourier transform ion cyclotron resonance (FTICR) MS and orbitrap MS are among the top choices (39). In addition to the separation and detection methods, the key bottleneck is the identification software. There are several welcomed free software and databases. ProSightP^TM^ (40) and TDPortal (41) might be the most widely used for TDP and proteoform identification, and TopPIC, TopMG, and Proteoform Suite might also be worthy of implementation (42). MetaMorpheus is an integrated software program for both BUP and TDP to identify peptides and proteoforms (43).

In addition to the mentioned whole-proteome strategy, the target-proteome strategy is sometimes preferred. An antiproteomics approach for the selection of nanobodies specific for overexpressed glioblastoma proteins was proposed recently (44). This straightforward antiproteomics approach led to the identification of seven novel candidate biomarkers for glioma formation, progression, and prognosis.

### Quantitative Proteomics

Quantitative proteomics can identify and accurately quantify proteins in biological samples and has become an effective research tool in the field of life sciences. Compared with the various oncogenes and tumor suppressors identified by genomics and transcriptomics research, the research objective of proteomics is the protein synthesized during translation. Proteins are the executors of most physiological processes; thus, through proteomics research, we can visually analyze physiological processes.

The isotopic-labeling strategies, in particular the isotope-coded affinity tag (ICAT), and stable isotope labeling by amino acids in cell culture (SILAC) were applied to evaluate metabolic marking proteins by using the principle of the dependence of mammalian cell proliferation on essential amino acids (45). Later, chemical labeling by isobaric tags for relative and absolute quantification (iTRAQ) (46), tandem mass tags (TMTs) (47), and dimethyl labeling (48) protocols were developed to improve quantification accuracy. These techniques use multiple stable isotope labels and amino groups of specific labeled peptides for tandem MS analysis. Although the quantitative information provided by SILAC and iTRAQ is considerable, the labeling reagent is relatively expensive, and the cost of each sample is large; therefore, these strategies are more suitable for quantitative analysis of protein expression changes at the whole proteomics level.

With the advancements in the field of proteomics technologies, it is now possible to measure an accurate amount of proteins in different biological specimens with label-free quantitative methods (49). Data-independent acquisition (DIA), is a remarkably developed label-free quantitative method in the past 5 years, which does not require expensive stable isotope labels as internal standards, but only needs to analyze the MS data generated with large-scale protein identification and compare the signal strength of corresponding peptides in different products to carry out relative quantitative analysis for proteins corresponding to peptide segments. DIA/SWATH-type techniques have been applied successfully in a variety of studies and are becoming increasingly prevalent in the quantitative proteomics field, especially in studies requiring consistent analysis of large sample cohorts, like the continuous collection of tumor samples for a long period.

Multiple reaction monitoring (MRM) is a targeted quantitative proteomics method to study target protein molecules, based on the information of target molecules. MRM MS is a high-precision protein quantitative identification technology, which is an excellent method for a one-time accurate quantitative study of multiple target proteins in complex samples.

### Single-Cell Proteomics

Analysis of single-cell transcriptomes using next-generation sequencing (NGS) has been intensively developed for decades. The methods to study glioblastoma multiforme (GBM) heterogeneity are mainly genetic and transcriptomic profiling, which cannot reflect instant functional changes (50, 51). Moreover, non-uniform results between genetic/transcriptome and protein levels have been shown, particularly for epidermal growth factor receptor (EGFR) (52, 53).

Recently, mass cytometry (MC) has become a more widely accepted platform for accurate proteomic analysis of single-cell dimension. MC is a technique proposed for the analysis of individual proteins in single cells. In this method, target proteins are quantified using antibodies conjugated with ions of isotopically pure transition metals. Protein complexes with the antibodies are sent through the inductively coupled plasma, which ionizes the metal conjugated antibodies, and their mass spectra are analyzed with a time-of-flight MS. MC has demonstrated the possibility of quantitative profiling of the immune response or evaluating the functional response of signaling at the single-cell level (54). This method has been increasingly used to analyze single cells when the research interest is focused on a limited group of proteins. Thus, MC was recently applied for quantitative analysis of transcription factors responsible for differentiation of hematopoietic cells (55).

Single-cell measurements, such as qFlow cytometry, provide a powerful tool to elucidate GBM heterogeneity. The fluorescent calibration is applied in qFlow to convert signal to accurate protein concentrations (56). Research on anti-VEGF efficacy based on qFlow cytometry and systems biology revealed that this efficacy is related to the concentrations of endothelial VEGFR1 in plasma membrane (57).

## Immunotherapy and Its Challenges

For malignant glioma, neuroimaging, surgical resection, radiotherapy, and chemotherapy are still standard care (58). In all grades of gliomas, surgical resection is necessary, and the maximal safe resection is still worthy to protect patients' neurological function (59). However, if the tumor is located in an important/non-resectable position of the brain and the tumor grows into the adjacent normal brain tissue, it is still difficult to completely remove the whole tumor. The highly specific and efficient method of immunotherapy is considered a promising therapy.

The immune system of patients with tumors is generally suppressed; thus, for the tumors with strong invasive ability, this feature makes it easier for them to become targets of treatment. Cancer immunotherapy (CIT) has developed fast in recent years and is increasingly playing an important role in cancer treatment. Tumor immunotherapy has shown a significant therapeutic effect in a variety of cancer types; thus, more and more research has focused on glioma immunotherapy. Immunotherapy can achieve a sustained response from the immune system without many side effects (60).

**Figure 2:**
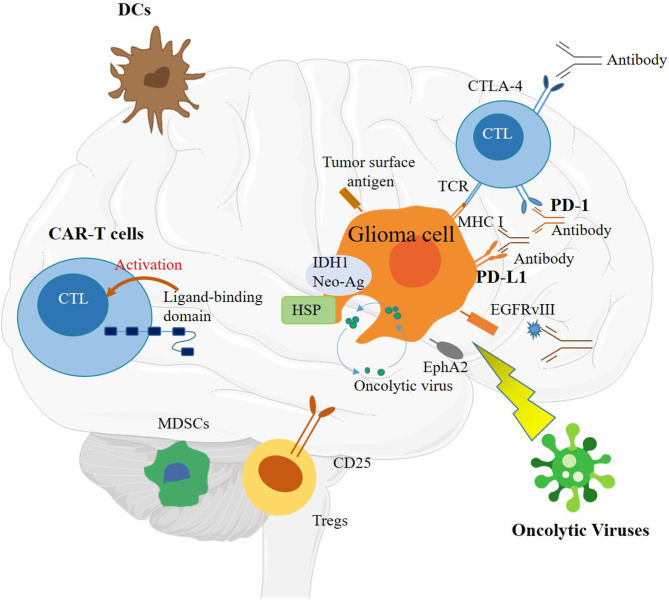
Schematic of immunotherapy, including immune checkpoint inhibitors, dendritic cell (DC) vaccines, chimeric antigen receptor (CAR) T cells, and oncolytic viruses (OVs).

Immunotherapy methods are currently under research and mainly including the following methods: peptide vaccines, oncolytic viruses (OVs), DC vaccines, CAR T cells, and immune checkpoint inhibitors (Figure 2) (58). However, there are still many challenges before these technologies can be applied. Although there are many successful applications of CIT on various human cancers, only a small number of patients benefit from these therapies. Specifically for glioma, the two important immune pathways have not shown many benefits (61). The main hurdles for immunotherapy in glioma include the low tumor mutational burden (TMB), heterogenetic microenvironment, restricted immune access, and sequestration of systemic T cells (58).

Among the challenges facing CIT, 10 top challenges were highlighted by Priti S. Hegde and Daniel S. Chen to promote cooperation (62). Here, based on the advantages of proteomics, we focus on the challenges in glioma related to heterogeneity and personalized biomarkers, driver mechanism elucidation, blocking points, combination of multiple immunotherapies, and prognostic evaluation methods (62). With the advantage of proteomics, the biotargets for immunotherapy and the mechanisms could be much more direct and easier to be discovered than the nucleic method. Like research in melanoma, the proteomic strategy has been a valuable platform for discovering novel biomarkers (63). Besides, the proteomics from different cells would be quite different which is even suitable for the research of heterogenetic characteristics. For now, the proteomics has been tried for the evaluation of therapeutic effects on glioblastoma (64).

### Monoclonal Antibodies Targeting Glioma Stem Cells

In immunotherapy research of glioma, the scheme of glioma-specific antibodies is also popular (65–71). It has been more than 30 years since monoclonal antibodies (mAbs) were used to target tumor antigens in immunotherapy. This scheme mainly depends on the antigens specifically expressed in glioma or the molecules overexpressed in tumor cells. mAbs have played an important role in tumor immunotherapy due to their direct cytotoxic and immunomodulatory effects (72).

Immunotherapy has been proved to be able to activate the antitumor response in the brain, which lays a solid foundation for treatment strategies for malignant glioma. The mAbs against PD-1 (nivolumab and pembrolizumab), PD-L1 (atezolizumab and durvalumab), CTLA-4 (ipilimumab) (58), EGFR (cetuximab) (73), and vascular endothelial growth factor (VEGF; Bevacizumab) (74) have been revealed with significant potential. A series of clinical trials of the mentioned targets are also underway (NCT02974883, NCT020177717, NCT01952769, etc.), but many challenges still need to be solved, such as the non-effect of anti-TGF-β antibody GC1008 on tumor progression in the late stage of treatment (75) and the non-survival benefit of bevacizumab (76). Additionally, methods to overcome the brain–blood barrier to deliver the mAbs to tumor sites should be developed, such as in the study of nimotuzumab, which targets the EGFR (77).

The recent cases have proven that proteomics studies could make some challenges easier to solve. Using proteomics, it is actually easier and more direct to detect and locate biotargets, namely, proteins. Differential expression brain-derived proteins, such as the EGFR, MMP9, TIMP, and fibulin-2 and−5, were validated to be released at the same time in one study of high-grade glioblastomas (19). The circulating biomarkers from the serum have been regarded as important sources for targeted therapy in brain cancers (78). A small panel of three proteins S100A8, S100A9, and CXCL4 were identified by proteomic strategy and validated by ELISA in early research (79). Another study to identify blood biomarkers also suggested another eight potential valuable ones, and three of them, LRG1, CRP, and C9, are closely related to the size of tumor (24). Different grades of glioma are analyzed by iTRAQ-based quantitative method, and it is found that nucleophosmin, glucose-regulated protein 78 kDa, nucleolin, and heat shock protein 90 kDa are highly expressed, and Raf kinase inhibitor protein is lowly expressed in glioblastoma. The expression levels of the RNA-binding protein nova 1 (NOVA1) in different subtypes of glioma were different (80). For all these proteins with altered expression, potential novel biotargets might be inside them. A series of proteins have been reported to have changes in their qualitative or quantitative composition during cancer development as determined with conventional methods (81, 82).

Bone marrow-derived human mesenchymal stem cells (BM-hMSCs) are expected to become cell vectors for glioma therapy due to their inherent glioma characteristics. Some GSCs are called attractors for they can attract the injected BM-hMSCs. The proteomics strategy could extend methodologies to further study various pathways related to inflammation-related cues for BM-hMSC homing (83). The results of the study present the first proof to link nutrition metabolism to N-glycosylation.

### Oncolytic Viruses

The abnormal expression of proteins in tumor cells caused by engineered oncolytic adenoviruses could be utilized to increase their anti-tumor efficacy. Oncolytic virus therapy is also a strategy for glioma immunotherapy. In addition to inducing cell death, virus infection can also cause endogenous and acquired immune responses, which are also promising immunotherapies. OVs are designed drugs that can selectively reproduce and kill tumor cells and then destroy the microenvironment of the tumor; thus, the innate immune system could be activated to adapt the immune response to tumor. It is an important design principle to weaken or delete viral virulence factors, making OVs safe for normal tissues, but still able to kill tumor cells in tumors (84). Delta-24-RGD (DNX-2401) and PSVRIPO are two promising engineered OVs resulting in better progress in clinical trials (85, 86).

For OVs, the current challenge is to understand the response mechanism of glioma cells to OVs, which will aid in the development of novel vectors with the stronger release of virus progeny to gain more effective oncolysis. DNX-2401, the E1A mutant of adenovirus, has shown proper toxicity and significant efficacy. Thus, the proteomics strategy and other techniques were conducted on cytosolic, nuclear, and secreted glioma proteomes to elucidate the interaction mechanism. The Delta-24-RGD can inhibit signal transducer and activator of transcription 3 (STAT3) and c-JUN (transcription factor AP-1), or increase nuclear factor kappa B (NF-κB) and protein kinase C (PKC), extracellular signal–regulated kinase 1/2 (ERK1/2) and p38 mitogen-activated protein kinase (p38 MAPK) pathways (87). Herpes simplex virus type 1 (HSV-1) is a vector with a great potential for application on solid tumors. The release of proteins is validated to be associated with metabolites, transportation, stress responses, apoptosis, proteolysis, the extracellular matrix, and cell adhesion by the proteomics analysis of HSV-1 infected human macrophages (88). In addition, filamin, tubulin, t-complex protein 1, and heat shock proteins are found to be upregulated, and extracellular matrix proteins are found to be downregulated by analyzing the secreted proteins and secretomes from tumor cells infected by oncolytic HSV-1 (89). These changes caused by HSV-1 RH2 infection indicated the potential to change the tumor microenvironment to improve the effect of immunotherapy.

### Dendritic Cell Vaccines

Tumor vaccines are an active form of immunotherapy, which can trigger the immune system to defend against tumors. The best way to activate the immune system is to stimulate dendritic cells (DCs), which are one kind of multifunctional antigen-presenting cells (APCs). The granulocyte-macrophage colony-stimulating factor (GM-CSF), interleukin-4 (IL-4), IL-1b, IL-6, prostaglandin E2 (PGE2), and tumor necrosis factor-α (TNF-α) mixture are utilized to stimulate peripheral blood mononuclear cells (PBMCs) to obtain mature DCs, which is the isolation source of DCs (90). The main purpose of DC vaccines to treat tumors is to generate specific T helper cells (Th) to activate the antitumor effect of cytotoxic T cells (91).

Though multiple glioma-related antigens, such as IL-13Rα2, HER2, EphA2, gp100, and aim-2, are targets of peptide vaccines and related clinical trials have shown that peptide vaccine treatment can also significantly prolong the survival period (NCT00643097, NCT00458601), the dominant drivers are still unclear, and the evaluation of prognosis is difficult. Even the driver scheme is complex, and proteomics could provide direct evidence to assist in discovery. The proteomics profile of tumor-associated macrophages (TAM) indicated that Cat Eye Syndrome Critical Region Protein 1 (CECR1) can promote differentiation of M2 TAMs and affects the proliferation and migration of glioma cells, and 67 proteins are upregulated by CECR1 siRNA transfection in THP-1-derived macrophages (MQs) (92). There have been studies based on proteomics to develop DC vaccines for solid tumors such as melanoma (93). The proteomics technique was applied to uncover the mechanism of how an original melanoma cell-derived lysate (TRIMEL) induced the immune responses mediated by T cell and DC maturation. Similarly, such an induced mechanism study could be applied in future glioma DC vaccine research.

### Chimeric Antigen Receptor T Cells

Cell adoptive T-cell therapy (ACT) has direct antitumor activity and could be developed as a personalized treatment. CAR T-cell therapy requires isolated T cells infiltrated by tumor from the patient's body. After stimulation with IL-2, it can be cultured *in vitro* to have the ability to specifically recognize the tumor and then returned to the patient (94). Unlike active immunity that stimulates the innate immune system with tumor-associated antigens, adoptively transferred CAR T cells can directly target tumor-associated antigens without relying on the antigen presentation process. CAR T cells have been successfully used in the treatment of hematological malignancies. This therapy targets EGFRvIII to clear tumor cells expressing EGFRvIII in tumor-bearing mouse models and phase I clinical trials (Trial No. NCT02209376) (95). However, due to the lack of specific antigens on the surface of solid tumors, the application of this therapy in solid tumors remains to be explored in depth (96). On the other hand, considering the adverse effects of CAR T-cell therapy on the CNS, such as cognitive dysfunction and hydrocephalus, there have been few reports on its use in gliomas (97).

In this immunotherapy area, advanced single-cell proteomics provides a more powerful method to evaluate the heterogenetic microenvironment. A study targeting on GBM39 indicated that over 70% target cells have more than 6,000 VEGFR2 (~five-fold higher) or PDGFRα/cell (~four-fold higher) plasma membrane proteins with higher expression levels (98). Within a 33-marker panel proteomics research, the complex immune microenvironments of single cell were illustrated, and the presence of various immune cells was confirmed. The increase of T cells with PD-1&CD8 or TIM-3&CD4 will induce the immunosuppressive effects in the microenvironments (99). A cohort analysis of 259 patients with primary and metastatic brain tumors ranging from benign to malignant by flow cytometry found that the myeloid-derived suppressor cells (MDSCs) of patients with GBM were significantly increased, which indicates a poor prognosis and provides a theoretical basis for formulating strategies for MDSCs (100).

As mentioned before, locating more biotargets for T cells to activate is also urgently need to apply CAR T-cell therapy. The reported 17 antigens with 41 different HLA ligands were identified through an MS analysis of HLA-presenting peptides in GSCs and glioblastoma patient specimens. Importantly, these become the best option for antigen-specific immunotherapy of glioblastoma for they are proved to be functional CD8+ T-cell epitopes in the tests of *in vitro* immunogenicity and killing antigen-specific target cells (101). In addition, comprehensive methods based on proteomics revealed that stable expression of GSC-specific antigen is related to higher T-cell infiltration and positive immunomodulator expression, indicating that the antigens are at reduced risk and suitable for further clinical application (102). The laboratory team of Sidi Chen applied membrane proteomics to update T cell–based immunotherapies (103). The detailed information validated that the edition of Pdia3, Mgat5, Emp1, or Lag3 genes in adoptively transferred CD8+ T cells can improve the survival rate of mice with syngeneic and T-cell receptor transgenic modification.

### Proteomics-Related Mechanisms to Assist With Immunotherapy

Mass spectrometry-based proteomics technology has not only started to contribute directly to immunotherapy but has also already aided to elucidate the signal and protein interaction mechanisms to improve the understanding of glioma diagnosis and molecular mechanism to assist the application of immunotherapy.

For glioma, the induction mechanism still needs to be clearly explained. Proteomics can currently cover a larger number of proteins and subsequently solidify the final drivers of glioma. Whole-genome sequencing and transcriptome sequencing only provide a hint of what is leading to the occurrence of the tumor; thus, the identification and quantification of specific proteins by proteomics could finally verify what is arising in the tumor to transfer the cells. Many large-scale proteomics studies have revealed that there are more possible candidate proteins to elucidate these mechanisms. A label-free quantitative proteomic study of low-grade astrocytoma (LGA) or GBM revealed 136 regulated proteins (86 up and 50 down) with at least a five-fold change in GBM (104). An unbiased quantitative proteomics analysis of human glioma biopsies revealed that up- or downregulation could be observed in multiple pathways. For instance, both clathrin-dependent and -independent endocytosis would be affected by a large reduction in various mechanical components related to the initiation, formation, and rupture of endocytic vectors, such as clathrin, AP-2 adaptins, and endophilins (105).

Beyond the whole proteomics comparison and filtering, more researchers have also applied proteomics to investigate specific pathways. The study of phosphorylated OLIG2 applies proteomics methods to reveal that glioma cells will have a stronger invasive mesenchymal character with the induction of non-phosphorylated OLIG2 to activate TGF-b2, providing a mechanistic insight for the transformation of cells from proliferation to invasion (22). The glioma cell line GL261 cultured with the 3T3-L1 adipocyte line verified that angiogenesis is necessary for adipose tissue expansion and is an important factor in the formation of malignant tumors (106) as well as in cancer progression and metastasis. Some identified factors from adipocyte cells are found underexpressed, such as STI1, hnRNPs, and PGK1 in conditioned glioma cells, and some are found upregulated in contrast, such as RFC1, KIF5C, ANXA2, N-RAP, and RACK1 in GL261 cell. In addition, pro-inflammatory and angiogenic factors are also with different regulations (107). A proteomics research on mouse glial culture indicated that glial cells will activate the MAPK/ERK pathway and upregulate a variety of proteins participating in inflammation, cell adhesion, and extracellular structural organization after exposure to GBM cells (108).

## Conclusion

For now, glioblastoma is one of the most lethal tumors due to its heterogeneity, which causes poor diagnosis and treatment. Even with multiple molecular markers, these complications make it hard to diagnose and classify cancer development and subtypes, even with the most advanced nucleic acid detection technology. Many novel technologies have been applied to mine more biomarkers to distinguish subtypes, and a series of new genes or proteins seem to be worthy of deeper research for both mechanism elucidation and identification of target therapies.

The standard treatments for tumors have not yielded much hope for patients. Significant progress has been made in developing immunotherapeutic regimens, and these may soon be included in the SOC. The development of immunotherapy is a valuable method to extend the lives of patients, but several challenges still need to be overcome. The tumors that do not respond to immunotherapy are often referred to as “cold tumors.” GBM is considered to be a cold tumor, and immunotherapy fails for many reasons, including the highly immunosuppressive tumor microenvironment, special physical microenvironment of glioma, and decreased tumor antigen presentation. The interaction of these factors together with the difficulty of T-cell activation recruitment and administration lead to the dilemma of immunotherapy for glioma. Thus, due to its complexity, the requirement to better elucidate the induction mechanism is urgently needed. Furthermore, the heterogenetic characteristics are a key challenge to identify more available biomarkers to activate the immune system. Additionally, similar to mechanism studies, the efficient observations of the therapeutic effect are another hurdle before clinical researchers. Many drugs and vaccines mentioned above might contribute considerably to other cancers but are still problematic for the treatment of glioblastoma due to the intratumor heterogeneity (109). The different combinations of multiple immunotherapies and standard care have also been evaluated by a series of clinical trials. However, locating more target positions might be more urgent and requires more devotion and better methodology.

Proteins can be used more feasibly than nucleic acids to assess the immediate situation of tumor development. Furthermore, most drugs have their final effect on proteins, which are the main life executors in the body compared with nucleic acids and metabolites. Thus, proteomics is a promising direction to mine more targets for diagnosis and treatment. The large-scale screening of quantity changes in proteomes makes it a more efficient filter for biomarker candidates. On the other hand, the heterogeneity of glioblastoma makes it more important to study several pathways simultaneously to unveil the mechanisms of occurrence, development, and immunosuppression.

## Future Prospects

With the enormous increase in the availability of gene expression, epigenetic and molecular pathway analyses, a personalized therapeutic approach tailored to the tumor would be ideal, especially for glioblastoma with intratumoral heterogeneity. With the rapid development of detection equipment and software, proteomics would be another promising and powerful tool to facilitate personalized therapeutics. The combination of multiple high-throughput technologies would enhance the progression rate of identifying more unique biomarkers.

With increasing research attention devoted to the application of proteomics for glioblastoma, more specific diagnostic procedures can be proposed based on MS detection. There might be four directions for the improvement of quantitative MS techniques to accelerate the application in biology and medicine: (1) updating and innovation of instrumentations, (2) optimizing sample preparation or fraction separation strategy, (3) developing more sensitive single-cell proteomics technology, and (4) developing more automated software tools. Though it is very exciting to be able to study proteomes, the next stage would be research on highly abundant proteoforms with large-scale analysis. Multiplexed proteomics technologies such as the reverse-phase protein arrays (RPPA) would also allow us to apply multi-omics, including genomic, transcriptomic, and metabonomic, to gain deeper understanding of tumor biology in the future.

## Author Contributions

LC, DQ, XG, and QW participated in writing the paper and figure preparation. JL guided the writing and editing of the article. All authors contributed to the article and approved the submitted version.

## Conflict of Interest

LC, DQ, XG, and JL were employed by the company ProteinT Biotechnologies, Co. Ltd., Tianjin, China. The remaining author declares that the research was conducted in the absence of any commercial or financial relationships that could be construed as a potential conflict of interest.
